# The Rock Island County Medical Society

**Published:** 1858-07

**Authors:** E. H. Bowman, W. A. Knox

**Affiliations:** President; Rock Island; Secretary; Rock Island


ROCK ISLAND, April 15th, 1858.

The Rock Island County Medical Society met in the City Council Room, Rock Island, on Wednesday, April 14th, 1858, the president, Dr. E. H. Bowman, in the chair.

Present, Drs. Gregg, Sharpe, Truesdale, Plummer, Cady, Hayes and Knox; also, Drs. Baker, Saunders and Shelton, of the Scott County Medical Society.

A communication from the Scott County Medical Society, inviting the Rock Island County Medical Society to attend their next meeting, to be held in Davenport, on the 27th inst., was read, and on motion, the invitation was accepted, and the communication placed on file.

Dr. Cady moved that a committee of three be appointed by the chair, to report resolutions expressing the sense of the society, in regard to the quack advertisements and patent medicine handbills now so extensively circulated, and the course of the druggists in relation thereto. The chair appointed Drs. Cady, Plummer and Knox.

Dr. P. Gregg was elected delegate to the American Medical Association, and Dr. Calvin Truesdale his alternate.

Drs. Sharpe and Plummer were elected delegates to the State Medical Society, and were authorized to appoint alternates if necessary.

Dr. J. R. Hayes read a very interesting essay on "Fever," for which the society passed a vote of thanks.

Dr. Sharpe reported a case of poisoning from oxalic acid, taken through mistake for sulphate of magnesia. Dr. Shelton reported a case of an attempt at suicide by swallowing a draught of nitric acid. Recovery followed a prompt and active course of treatment in both instances.

Drs. Gregg and Baker related several remarkable cases of recovery following penetrating wounds of the chest.

On motion, the proceedings of the society were ordered to be sent to the *Chicago Medical Journal* for publication.

Society then adjourned.

E. H. BOWMAN, *President.*

W. A. KNOX, *Secretary.*

